# AI-Augmented Clearinghouse to Facilitate Evidence-Based Decision-Making and Social Spending: a Conceptual Framework

**DOI:** 10.1007/s11121-025-01848-1

**Published:** 2025-10-11

**Authors:** Pamela R. Buckley, Diana Fishbein, Neil J. Wollman

**Affiliations:** 1 National Prevention Science Coalition to Improve Lives (NPSC), Chapel Hill, NC USA; 2 Frank Porter Graham Child Development Institute, University of North Carolina, 910 Raleigh Road, Chapel Hill, NC 27559 USA; 3 Human Development and Family Studies, The Pennsylvania State University, University Park, PA USA; 4 Blueprints for Healthy Youth Development, Boulder, CO USA; 5 University of Colorado Boulder, Boulder, CO USA

**Keywords:** Artificial intelligence, Clearinghouse, Preventive interventions, Implementation, Evidence-based

## Abstract

Social spending refers to public or private funds aimed at helping vulnerable groups. Evidence-based decision-making supports social spending by applying existing evidence to inform funding and involves integrating relevant information from various sources, such as experimental studies and community feedback. Online clearinghouses support evidence-based decision-making by synthesizing evidence on what works, though manually updating literature is incomplete. In addition, passively summarizing evaluations is insufficient for end-users to implement preventive solutions that achieve population impacts. A responsive platform and a dissemination plan are needed to encourage the uptake of equitable and culturally relevant preventive interventions grounded in transparency and rigorous evidence of effectiveness. A two-part conceptual framework is proposed for a clearinghouse platform that integrates stepwise, interactive, AI-driven capabilities safeguarded by human touchpoints. First, clearinghouses should adopt a “living” review to automatically update evaluations—an approach embraced globally by organizations such as the World Health Organization, Cochrane Collaboration, and the U.N.’s Pan American Health Organization. The second component involves adding a chatbot to support assessment and implementation guidance and make recommendations on (1) the provision of all evidence-based preventive interventions (EBPIs) and their key activities, (2) EBPIs shown to achieve equitably distributed outcomes, (3) culturally relevant EBPIs that align with the values and needs of a target population, (4) implementation support, such as materials, training, and fidelity measures, and (5) delivery costs. The resulting platform will ethically expedite the translational process of identifying and scaling EBPIs, leading to a more complete, comprehensive, and accessible body of evidence on effective preventive strategies.

**Supplementary Information:**

The online version contains supplementary material available at https://doi.org/10.1007/s11121-025-01848-1.

Social spending refers to public or private cash transfers (such as grants, contracts, and donations), in-kind services, and tax incentives aimed at supporting vulnerable groups—including children, the elderly, people with disabilities, and households from low-income backgrounds—through physical, mental health, and education programs (among others). Scientific evidence is a critical tool for informing social spending, as it reveals what works, in what settings, and for which individuals, schools, and communities (Fishbein et al., 2016). Evidence can protect taxpayer and philanthropic interests by informing decision-makers about where best to invest money to ensure people who need help can secure it and to drive faster progress, thereby maximizing the ability of government funds and charitable contributions to improve lives (Ellor, 2024). Evidence also identifies strategies most effective for specific populations and can therefore be a powerful tool for reducing disparities. And it can detect preventable differences in health outcomes, leading to approaches for achieving optimal health between groups of people. By focusing efforts on programs shown to promote health equity—which implies that everyone has a fair opportunity to attain their full health potential—evidence can also help ensure that resources are allocated where they will have the greatest impact (Braveman et al., 2018; Buckley et al., 2025).

Evidence-based decision-making emphasizes the use of data and rigorous findings of the best available evidence intersected with decision-makers’ expertise, community needs, and implementation context to inform social spending. It involves systematically gathering, evaluating, and synthesizing relevant evidence from various sources, such as scientific studies, experimental data, expert opinions, and community feedback (Baba & HakemZadeh, 2012). Availing ourselves of opportunities for evidence-based decision-making to play out can be particularly impactful with the application of evidence amassed in the field of prevention science, which directs us to well-tested programs and policies shown to interrupt pathways to negative outcomes and improve the behavioral health and wellbeing among our children, youth, families, and communities (Fishbein & Sloboda, 2023).

Results for America is a nonprofit organization in the USA that helps government leaders harness data to solve challenges. The organization released a report detailing bipartisan progress made over the last decade to steer more dollars to evidence-based decision-making and help evaluate what works, including the US Foundations for Evidence-Based Policymaking Act of 2018 that created a framework for evidence-building (Ellor, 2024). Private foundations such as Arnold Ventures (in the USA) and the Wellcome Trust (in the UK) are also encouraging states and counties to take a more evidence-based approach to supporting behavioral health services. Despite these advances, however, rigorous evaluation requirements have so far gained only a foothold in social policy, and most public and private spending (both in the USA and internationally) is still allocated with little regard to evidence about what works (Axford et al., 2022; Baron, 2018). The challenges have been in increasing the utility and awareness of what works, for whom, and in what settings so that this information can be more accessible, applicable, and equitable for end-users such as communities, schools, agencies, funders, and policymakers.

To overcome these challenges, online clearinghouses were developed to provide free web-based syntheses of the evaluation literature by rating interventions based on their level of effectiveness to inform decision-making by communities, funders, policymakers, and practitioners (Buckley, 2024; Lee et al., 2022; Lee-Easton et al., 2022; Means et al., 2015). Clearinghouses, however, require significant human effort to screen and organize studies, which can delay the timely availability of evidence. In addition, users have unidirectional interactions with online clearinghouses, which limits their utility. And online clearinghouses rely on word-of-mouth advertising, grant solicitations, e-newsletters/listservs, presentations, and booths at conferences, journal articles, and links on websites to promote use, rather than an explicit marketing plan to broaden dissemination and use (Burkhardt et al., 2015).

This paper outlines a two-part conceptual framework describing how online clearinghouses can leverage artificial intelligence (AI) and related technologies to strengthen evidence-based decision-making and social spending by accelerating the translational process of identifying and scaling evidence-based interventions. Included in this conception is a large-scale outreach campaign to substantially increase the reach and salience of an AI-augmented clearinghouse platform to a broader range of constituent groups. An example of how AI can be built into a clearinghouse to specifically support the prevention science field is exemplified using Blueprints for Healthy Youth Development, i.e., Blueprints (Mihalic & Elliott, 2015), the longest-standing online clearinghouse (launched in 1996) and the only one solely focused on platforming evidence-based *preventive* interventions (Buckley, 2024).

## The Evidence Ecosystem

An “evidence ecosystem” encompasses the complex network of individuals, organizations, processes, and knowledge that work together to generate, disseminate, and apply reliable evidence to inform decision-making. The AI-augmented conceptual framework described herein is situated within the broader context of reductions in this evidence ecosystem, particularly in the United States with the termination of funds starting in January 2025 for research and a reduction in force (RIF) of experienced federal workers that administered many of these clearinghouses and produced a range of evidence-based resources (e.g., evidence standards, implementation guidelines, and intervention reports).

Online clearinghouses provide a free resource to support social spending and the public good, and thus are funded by a range of sources, including governmental agencies, nonprofit organizations, and international organizations (Burkhardt et al., 2015). Because online clearinghouses rely on grants and government contracts, they are highly vulnerable to fluctuations in funding priorities from both public and private sources. Shifts in leadership can lead to the reallocation of resources, changes in policy focus, or outright discontinuation of grants that do not align with new priorities. For example, the National Registry of Effective Prevention Programs (NREPP) was an online clearinghouse of interventions designed to prevent or treat substance abuse and mental disorders that was funded and administered by the Substance Abuse and Mental Health Services Administration (SAMHSA) within the US Department of Health and Human Services (USDHHS). NREPP was suspended in 2018, primarily due to concerns over its methodology and shifting priorities within the USDHHS (Green-Hennessy, 2018). More recently, in February 2025, the US Department of Education abruptly canceled numerous contracts with the Institute of Education Sciences (IES), which funds the What Works Clearinghouse (WWC), focused on teaching and student learning. Before the contract cancellations, however, IES was exploring ways to modernize the WWC, including recommendations for the implementation of living evidence reviews (discussed later; Anderson et al., 2025). These realities are problematic for several reasons, notably that federal, state, and philanthropic decision-makers rely on clearinghouses to de-risk spending (i.e., invest in effective programs, avoid harmful ones). If review pipelines are hindered, procurement decisions revert to marketing claims or dated syntheses, which raises the odds of waste and potential harm.

The precarity of research infrastructures—including the evidence ecosystem that supports social spending—highlights the need for a resilient framework that can withstand shifts in the political and economic climate. Securing diversified funding sources, establishing transparent governance structures, and ensuring broad stakeholder engagement protect the sustainability of evidence review mechanisms in the future. It also speaks to the importance of innovation to ensure productivity, reliability, utility, and sustainability. In the evolving landscape of social spending, maximizing the impact and efficiency of evidence-based decision-making is paramount. Online clearinghouses serve as valuable resources for identifying and promoting effective interventions. Modernizing their features and incorporating advanced technologies such as artificial intelligence (AI) can dramatically enhance their value, drive demand, and help sustain these platforms despite the challenges of fundraising for research infrastructure.

## Online Clearinghouses in Present Form

Online clearinghouses are one tool for identifying and disseminating information on what works for whom and in what contexts. They serve as repositories of reports synthesizing the effectiveness of behavioral interventions for infant, youth, and adult populations that fall under a wide range of disciplines and domains, such as criminal justice, child welfare, public health, mental health, education, and labor/employment, among others (Buckley, 2024; Horne, 2017; Neuhoff et al., 2015). These clearinghouses translate the evaluation literature and raise awareness about the existence of evidence-based interventions (Buckley et al., 2020).

### Current Functions

To identify which interventions are “evidence-based,” online clearinghouses review intervention evaluations (i.e., primary studies) using published standards of evidence focused primarily on internal validity and evidence of effectiveness supported by randomized controlled trials (RCTs) or other quasi-experimental design studies (QEDs) using causal inference methods. They require, for example, that intervention and control groups are comparable at baseline, attrition is minimal and not differential, and outcomes are assessed using reliable and valid instruments (Wadhwa et al., 2024; Zheng et al., 2022). Clearinghouse procedures typically involve (1) identifying RCTs and QEDs of programs through literature searches; (2) assessing each study’s significance and internal validity—risk of bias assessment (Steeger et al., 2021); (3) representing evidence in a text form that is comparable across studies; (4) synthesizing evidence across evaluations by writing extensive and detailed reviews; and (5) presenting the synthesis online in (ideally) readily understandable and usable ways (Axford et al., 2022; Buckley, 2024; Buckley et al., 2020, 2022). To ensure reliability, clearinghouses make decisions (i.e., #2 above) based on multiple raters with knowledge of evaluation methods, provide raters with training in the standards used to determine effectiveness, and require the use of structured rating instruments (Buckley et al., 2020; Steeger et al., 2021).

These clearinghouses provide a wealth of information, such as a description of the intervention and an effectiveness rating designed to allow users with minimal knowledge of research methods to select an evidence-based intervention or to vet those that have been adopted. Additionally, some online clearinghouses provide a written summary of costs, training, and/or the availability of technical assistance to assist with dissemination and scaleup (Buckley et al., 2020; Paulsell et al., 2017). In providing electronic publication of evidence reviews, online clearinghouses: (1) are not constrained by lack of space; (2) can be updated as new information becomes available or when new ways of improving them are identified; and (3) can be cross-linked to other, related sources of relevant information (Starr et al., 2009).

### Proliferation (from 1996 to 2024)

The number of online clearinghouses has grown prolifically over the past 30 years, many in response to governing bodies requiring evidence to support funding decisions and limit unnecessary spending (Buckley, 2024; Horne, 2017; Lee et al., 2022). As of December 2024, up to 24 clearinghouses existed within the United States and Europe alone (Axford et al., 2022; Mayo-Wilson et al., 2022). Research shows minimal overlap across US clearinghouses in terms of the individual interventions reviewed (Wadhwa et al., 2024; Zheng et al., 2022), reflecting a need for the number of clearinghouses given the vast amount of evidence being generated by the social sciences. Consistent with other fields (e.g., medicine, physics, etc.), users cannot keep up with this volume of work, and many consumers do not have the time or expertise to assess which studies are of high quality and worthy of consideration when making decisions. Clearinghouses are, therefore, increasingly important as the volume of relevant evidence expands, and with widely varying quality of the studies, even those in the peer-reviewed literature (Buckley, 2024).

A challenge remains, however, in providing sufficient resources and support for users to meaningfully engage in the use of research evidence (Mosley, 2023), including clearinghouse reviews. Although online clearinghouses were designed to simplify the task of selecting evidence-based interventions, their expansion has caused confusion stemming from how they differ in (1) focus (i.e., one domain—such as the US Department of Education WWC’s focus on student learning—versus multiple domains, such as Blueprints’ focus on healthy youth development); (2) modality (program, practice, or policy); (3) accepted designs (meta-analysis, only RCTs, certain QEDs, or no restrictions); (4) benchmarks for when “slippages” in evidence standards (e.g., baseline equivalence and differential attrition) bias results; and (5) ratings, wherein some clearinghouses rate interventions as “evidence-based” or not, while others rate interventions according to several “tiers” of evidence that explicitly require, for example, replication and/or sustained effects for a top tier rating (Buckley, 2024; Wadhwa et al., 2024; Zheng et al., 2022). Although it is unclear why online clearinghouses rely on different standards, criteria are likely influenced by political concerns, scientific debates and ideologies, and financial and other practical considerations (Fagan & Buchanan 2016; Means et al., 2015). In addition, online clearinghouses update their websites on different schedules, likely because the process for identifying and reviewing programs is laborious and expensive, causing concern about the timeliness of generating evidence reviews (Means et al., 2015). These challenges contribute to a messy evidence landscape and result in different levels of confidence regarding the effectiveness of interventions recommended for scale-up (Axford et al., 2022; Zheng et al., 2022). Thus, clearinghouses must clarify inconsistencies to reduce user confusion, standardize procedures to the extent possible, *and* simultaneously innovate to ensure their feasibility and relevance.

### Limitations

In addition to collective user confusion as outlined above, several shortcomings of individual online clearinghouses were outlined in the Bridgespan Report on a study of the “What Works Marketplace” (Neuhoff et al., 2015). Accordingly, clearinghouses are not as widely utilized as the evidence warrants, limiting their impact. First, while Lee et al. (2022) found that important groups utilize certain clearinghouses, and that these clearinghouses are useful to the types of users (e.g., grant writers, practitioners, and some agency directors) who access them, online clearinghouses are not easily navigated by most end-users (e.g., community groups, practitioners, state agencies, policymakers). The literature (while limited) suggests that online clearinghouses are not well understood or accessible, especially by regulators charged with making policy (Lee et al., 2022). Second, clearinghouse administrators are required to routinely manually update the literature—a process that is inefficient and incomplete. Third, passively viewing evaluation summaries is not an effective vehicle for transmission to communities, policymakers, and other decision-makers (Lee-Easton et al., 2022). Currently, users have only unidirectional access. That is, filters defined by clearinghouse staff allow users to search for interventions based on outcome (e.g., substance use, delinquency, academic achievement, emotional well-being, physical health), target population (e.g., age, sex, gender, race or ethnicity), and program specifics (e.g., program type, program setting). Users click one or more of these filters to generate a list of interventions and studies reviewed that meet all the criteria of these pre-defined filters; however, there is no bidirectional interaction to reach an optimal solution. And fourth, as mentioned previously, clearinghouse reviews generally focus on internal validity (the ability to make causal inference; Steeger et al., 2021; Zheng et al., 2022); however, they lack information on how to consider transparency, cultural context, and health equity (Buckley et al., 2022, 2023, 2025; Mayo-Wilson et al., 2022; Newcomer et al., 2023).

Given growing concerns related to the applicability of causal inferences, users want information on the reporting of health equity in RCTs (e.g., whether benefits are concentrated among those who are already advantaged) and cultural relevance (i.e., how well the intervention aligns with and respects the cultural beliefs, values, practices, and needs of a target population; Newcomer et al., 2023; Strayhorn et al., 2024). They also want the ability to identify innovative and community-developed programs that have yet to be subjected to rigorous evaluation. And having open access to the research materials, the anonymized data collected, and the code used to analyze data is valuable—as transparency and reproducibility have long been recognized as vital functions of science (Buckley et al., 2022; Mayo-Wilson et al., 2022).

## Improving Clearinghouse Functionality Via Responsible AI

To address clearinghouse shortcomings and gaps in awareness of the vital resource offered by online clearinghouses, a responsive and comprehensive literature review process, platform, and dissemination plan are needed to encourage the uptake of interventions grounded in rigorous evidence of effectiveness. Responsible artificial intelligence (AI) is a valuable technology to facilitate the process of identifying, selecting, and implementing evidence-based interventions for greater accessibility, instructiveness, and reach by end-users (e.g., practitioners) and, in turn, improving investments by funders, including philanthropists, policymakers, and taxpayers. Broadly speaking, AI—which dates back to a summer workshop at Dartmouth College in 1956—refers to the efforts of computers in mimicking human problem-solving abilities (Howard, 2019; Voss, 2024). *Responsible* AI refers to the development and deployment of AI systems that are transparent, fair, accountable, and aligned with ethical, legal, and societal values, ensuring that potential risks are anticipated and mitigated while benefits are equitably distributed (Floridi et al., 2018). Below is an overview of features enabled by responsible AI that can improve the functionality of clearinghouses and promote evidence-based decision-making.

### Living Systematic Review

A relatively new synthesis method is called a “living” systematic review, wherein research articles and reports are updated as new findings become available and relevant evidence is incorporated into the review. Unlike traditional systematic reviews, which are typically static and updated manually at unspecified intervals or after long periods of time, living evidence reviews update evidence in shorter, predetermined intervals, allowing the public to access current findings on a given topic (Brooker et al., 2019). Within the living evidence review approach, various AI tools are used to automate the monitoring of new research publications, such as web scrapers, RSS feeds, or automated alert systems (like PubMed and Google Scholar alerts) that index new studies related to specific topics. Meanwhile, machine learning can assist in the identification of relevant studies and extract data from publications, which helps in scaling the review process and increasing the comprehensiveness of information included in the review. Several recent papers provide an overview of the emerging living systematic review concepts, noting key challenges as well as the strategies and potential of a living approach (Cheyne et al., 2023; Navarro et al., 2023; Turner et al., 2023). Living evidence has been embraced globally, with the World Health Organization, Cochrane Collaboration, and United Nations’ Pan American Health Organization all committing to adopting this approach (Chakraborty et al., 2024).

### AI Chatbots and Large Language Models

A Large Language Model (LLM) is a type of AI chatbot that is designed to understand, generate, and interact with human language. LLMs are part of the field of Natural Language Processing (NLP), a branch of AI focused on the interaction between computers and human language. NLP includes tasks like understanding and generating text, translating languages, answering questions, summarizing content, and more. One such model, ChatGPT (Generative Pre-trained Transformer), is a human preference-aligned chat bot from Microsoft-backed OpenAI that has garnered significant attention by the public since its launch in November 2022 (Scarlatos, 2024). ChatGPT is the equivalent of a giant library where LLMs (which are the core of ChatGPT) have read all available texts and learned patterns in how words and sentences are formed to predict the next word or sentence. So, when a user asks ChatGPT a question, ChatGPT predicts the most likely response based on its knowledge of language and patterns it learned from all the information it processed. ChatGPT therefore engages users in interactive dialogue, encouraging them to ask questions and explore topics more deeply (Alghizzawi, 2024; Gartlehner et al., 2024). This feature could facilitate evidence-based decision-making by training LLMs on all available information within and across clearinghouse platforms and providing more personalized assessment and implementation guidance for clearinghouse users. The advantages of leveraging AI technologies such as ChatGPT, however, need to be considered along with potential risks associated with this evolving technology (Greenstein & Cho, 2025).

## AI Risks

For all the potential benefits AI poses, the risks could cause more harm than good in the absence of proper safeguards. For example, these AI models can make factual errors (i.e., “hallucinations”) that result in false, unvetted, or misleading information. In addition, responses lack real-time expertise and accuracy. As a language training model, ChatGPT does not possess an understanding of the knowledge it produces. ChatGPT has a natural advantage in content generation, as it autonomously creates conversational content based on user prompts, bypassing the overreliance on the traditional knowledge of professionals. However, its ability to generate responses relies on a large amount of “input data,” which may contain biases and prejudices due to historical inequalities and inaccuracies, leading to the mischaracterization of certain demographic groups which serve to further health disparities or lead to social tension. There is also the potential for a lack of transparency regarding the sources of data or specific conclusions, which can undermine public trust (Slattery et al., 2024; Zhang et al., 2024).

And while AI has the potential to transform many aspects of society, it can also exacerbate inequities if not implemented with careful consideration of these differences. Specific to the prevention science field, dominant paradigms may be amplified by AI, especially in terms of which programs are represented, how they are interpreted, and whose perspectives are excluded. Recent studies suggest insufficient evidence exists of preventive programs evaluated with a RCT and validated with populations from historically marginalized communities. Buckley et al. (2023) synthesized 885 preventive programs with experimental evaluations from 2010–2021, finding that not all studies reported race or ethnicity. Of the 77% that included race, samples were White (35%) or Black or African American (28%), and of the 67% that reported ethnicity, Hispanic or Latino participants were represented in 32% of the samples. American Indian or Alaskan Native, Asian American, and Native Hawaiian or Pacific Islander populations were largely absent or grouped into an “other” category. Most studies reported gender in binary terms, about one-third included rural participants, but fewer than one-third reported socioeconomic background. Although clearly indicated, a follow-up synthesis of 292 effective preventive programs found few culturally grounded interventions, with only 4% designed for African American or Black populations and 1% for Hispanic or Latino youth (Buckley et al., 2025). Subgroup analyses testing “for whom” programs “worked” were in short supply, with 25% testing effects by race, 15% by ethnicity, 28% by economic disadvantage, 47% by binary gender, and virtually none by location (rural or urban). Drawing from Buckley et al., (2023, 2025), AI-augmented clearinghouses requiring the use of experimental evidence may inadvertently risk perpetuating inequities if widely used programs tested for one group are applied, perhaps uncritically, to others (Thier et al., 2020).

The possibility of job displacement is another crucial consideration in the growing reliance on AI. These risks include job loss, deskilling, or devaluation of experiential knowledge. As AI tools increasingly dominate workplaces, irrespective of their purpose, time will tell which way the balance tips: toward job creation or reduction (Howard, 2019). Employers, AI developers, and users should actively monitor changes and make necessary adjustments to prevent worker displacement. Policies to address potential job loss are still being considered. The next section suggests strategies that may apply specifically to AI-clearinghouse development.

## AI Safeguards

Platforms that adopt AI must take these risks into serious consideration and develop protection to guard against AI’s potential negative unintended consequences. Ensuring responsible AI requires both *technical* and *procedural* safeguards. Examples of technical measures include (1) audit trails (i.e., creating records of all inputs, outputs, and model changes to allow for tracking the decision-making process and identifying potential biases); (2) bias detection algorithms, which are algorithms designed to identify and quantify bias in AI models to help ensure fairness and prevent discriminatory outcomes; and (3) diverse data collection practices (i.e., gathering data from a wide range of sources and demographics to train AI models on representative datasets that are less likely to reflect biases). Examples of procedural safeguards include: (1) community review boards that involve diverse constituents in the review and validation of AI systems to ensure that the system is aligned with ethical and societal values; (2) human-in-the-loop validation, which incorporates human oversight into AI decision-making to allow for a second layer of review and help mitigate potential biases or errors; and (3) stakeholder engagement, which involves actively soliciting feedback from end-users, ethicists, and domain experts throughout the AI development process to ensure that the system is aligned with real-world needs and ethical considerations (Al-Saadi, 2025; Lewis et al., 2024).

To address epistemic bias, including the representation of populations from historically marginalized communities in study samples and relegation of culturally grounded evidence, AI can facilitate the inclusion of innovative and community-developed programs that have yet to be subjected to rigorous evaluation involving a QED or RCT. A process for their review would need to be developed to (1) denote their stage of development (for an example, see Buckley, 2025), and (2) indicate whether further evaluation is needed to meet clearinghouse effectiveness standards.

Regarding the impact of AI on the workforce, AI should be used as a force-multiplier for public servants, researchers, and staff who oversee the evidence ecosystem, not a substitute. That is, wholesale automation magnifies risk (i.e., service gaps, errors, and inequities). A “workforce-first” AI policy flips the incentive: it can amplify human capacity rather than cut it. Protecting personnel from the risks involves ensuring human oversight of AI decisions to prevent “automation bias” (i.e., blind trust in AI output), training staff in AI tools, and developing clear policies for defining how AI offloads low-value tasks and elevates professional judgment. These principles can be codified via workforce-protection policies, independent audits, and human-in-the-loop controls. In addition, “savings” from augmenting human decision-making with AI tools can be reallocated to training, community engagement, and quality assurance (Howard, 2019).

## AI Governance

AI presents a distinctive governance challenge. While its reach is undeniably global, it also has localized impacts, influencing individuals, families, workplaces, and communities in ways that can differ greatly. Discussion of who will exert control and oversight over funding and use of AI in evidence-based decision-making is relatively unexplored. Hence the purpose of this paper, which presents the opportunities for advancement via responsible AI, while also calling attention to the issues and areas of concern that require further address.

Under consideration across fields are several government and regulatory bodies that could play a governance role to ensure responsible AI; e.g., Office of Management and Budget or the Office of Science and Technology Policy. However, in the current political environment (i.e., as of 2025), implementing these safeguards in an ethical, transparent, and scientifically rigorous manner cannot be assured. Another possibility is for industries to establish self-governance protocols, although these should be complemented by robust external oversight mechanisms to ensure accountability and transparency. By developing clear standards for responsible AI adoption and regularly evaluating their effectiveness, industries can mitigate risks and maximize the benefits of emerging technologies. It is also possible for states to provide effective oversight; however, the consideration here is that enactment is likely to significantly differ across states with varying political and policy agendas. A more scientifically palatable approach may be establishing infrastructure for independent bodies and scientific panels, all with the need for vehicles to obtain community input. Such collaborative efforts, supported by technical and procedural safeguards as outlined above, have the potential to foster ongoing innovation while safeguarding against unintended consequences. Integrating these approaches into clearinghouse platforms will further strengthen evidence-based decision-making and lay the groundwork for more adaptive and trustworthy systems that effectively serve end-users and the wider public.

## Exemplifying Utility of AI With a Well-Known Online Clearinghouse

Like other clearinghouses, Blueprints for Healthy Youth Development (“Blueprints”; Mihalic & Elliott, 2015) synthesizes evidence-based interventions, including programs, practices, or policies grounded in empirical evidence that support their efficacy, effectiveness, and safety (Gottfredson et al., 2015). Blueprints, however, is the only online clearinghouse solely focused on synthesizing evidence-based *preventive* interventions (EBPIs), which share attributes of evidence-based interventions. The term “preventive”, however, underscores their proactive nature. Also referred to as “upstream preventive programs,” EBPIs intervene before problems occur or escalate to reduce the burden of disease and dysfunction on individuals, families, communities, and societies (Fishbein & Sloboda, 2023; Fishbein et al., 2016).

Although the framers of Blueprints regularly conduct upgrades, there is a need now to functionally advance and address many of the same limitations of other clearinghouses to deliver on their purpose, as outlined by the National Prevention Science Coalition to Improve Lives (https://www.npscoalition.org/ebp-clearinghouse-proposal). In this example, we present a plan to continuously update and systematically appraise Blueprints’ summaries of preventive intervention research evidence using responsible AI. The Blueprints’ database could also be linked to networks of scientific partners who collectively disseminate resources on identifying, testing, and scaling preventive interventions to outline the data infrastructure and its features. The result is a one-stop resource for current information and assistance needed to effectively deploy a range of evidence-based preventive strategies. We describe how new technologies and real-time, transparent, equity-centered approaches can be leveraged to connect prevention science and implementation science within one AI-augmented closed (and trustworthy) system to meet decision-makers’ needs for a more thorough suite of effective preventive strategies.

### Blueprints’ Current Operational Model

To understand how AI can enhance Blueprints’ functionality, we first summarize the process of Blueprints’ systematic reviews and features that allow for user interface. Blueprints’ standards include four criteria, which are consistent with evaluation guidelines recommended by the Society for Prevention Research (Gottfredson et al., 2015). They include: (1) Intervention specificity—all programs must clearly identify the theory(ies) guiding the intervention, their targeted risk and protective factors and outcomes, the populations to be served, and their specific delivery methods. (2) Evaluation quality—studies must implement a strong group design and sound statistical analyses. Blueprints’ staff and advisory board conduct a risk-of-bias assessment to highlight potential flaws that could bias results. (3) Intervention impact—the preponderance of evidence must show consistent, statistically significant, and positive effects on outcomes. (4) Dissemination readiness—programs must be available for use with instructions (e.g., manuals, training, etc.) to guide implementation, including costs and an explanation of staff resources to deliver the intervention. Online Resource Table 1 lists Blueprints’ current evidence criteria.

## Enhanced Clearinghouse Functionality Via AI

**Fig. 1:**
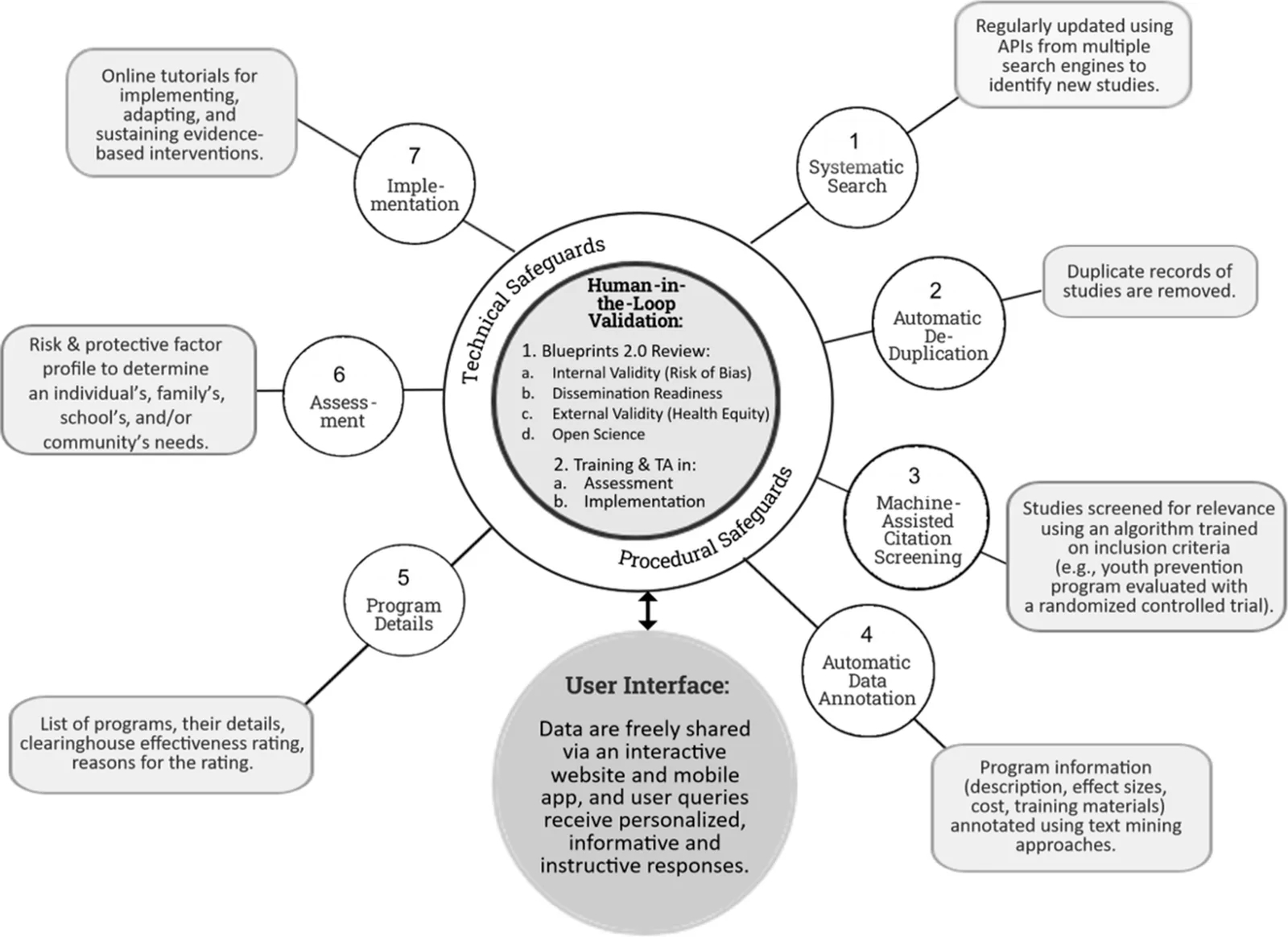
AI-augmented clearinghouse framework. Notes: Central to this framework are safeguards to mitigate bias and misinformation. Part I: living evidence review to identify and annotate studies (1–4); Part II: AI chatbot to support assessment and implementation (5–7). Visual partially adapted from Butler, A. R., Hartmann-Boyce, J., Livingstone-Banks, J., Turner, T., & Lindson, N. (2024). Optimizing process and methods for a living systematic review: 30 search updates and three review updates later. *Journal of Clinical Epidemiology, 166*, 111231

Herein, we propose a two-part framework to modernize clearinghouses—which describes Blueprints 2.0—or the next generation of AI-augmented clearinghouse platforms (Fig. 1).

### Part 1: Living Evidence Framework to Automatically Identify and Annotate Studies

The first part of this Blueprints 2.0 framework (that could apply to *any* online clearinghouse) involves harnessing machine learning to create a “living evidence” framework to enhance the efficiency and reliability of literature search and data extraction processes to ensure that the clearinghouse database is continually updated (Gartlehner et al., 2024; Turner et al., 2023). Large Language Models (LLMs) could be used to increase the efficiency of the literature search strategy, replacing the manual process of: (1) entering multiple search terms into individual search engines and individually reviewing abstracts for eligibility, (2) searching blogs, other clearinghouses, and web pages of research organizations to identify additional studies, and (3) accepting nominations from developers and/or researchers. This activity not only facilitates the process of garnering and organizing a large body of information, but it also significantly enhances the comprehensiveness of that information. In addition, Natural Language Processing (NLP) could be incorporated into the labor-intensive process of manually extracting data from primary studies into standardized tables. For example, NLP algorithms could extract from reports and articles the intervention’s description, study setting, sample characteristics, outcomes, and effect sizes. These algorithms could also replace labor-intensive queries to examine the readiness of the intervention for dissemination by extracting publicly available information on materials, training, and costs, including policies and management systems to be in place for the intervention to be implemented with fidelity. Following “human-in-the-loop validation”, researchers could check the extracted data to ensure accuracy, completeness, and consistency (Gartlehner et al., 2024). In addition, the Blueprints staff and board could also then conduct their standard risk-of-bias assessment (Steeger et al., 2021), for which NLPs could be used to efficiently expand the Blueprints 2.0 evidence review criteria to also consider criteria assessing health equity and cultural relevance of interventions (Buckley et al., 2023, 2025; Newcomer et al., 2023) and transparent evaluation methods, such as preregistration and sharing of data, code, and research materials (Buckley et al., 2022; Mayo-Wilson et al., 2022). See Online Resource Table 1 for an explanation of these expanded criteria.

### Part 2: AI Chatbot to Support Assessment and Implementation Guidance

The second component of this Blueprints 2.0 framework involves adding a chatbot search engine that would provide for the type of interaction via AI software (e.g., ChatGPT) that might occur in a conversation, where one statement or query leads to a more personalized, informative, accurate, and instructive response for providing contextual and operational follow-up. ChatGPT technology could serve multiple functions. First, ChatGPT could play an integral role in helping users navigate clearinghouses like Blueprints by allowing for conversations with human-like responses and interactions (Alghizzawi, 2024). It can also be used to automate various clerical tasks performed by clearinghouse staff (e.g., responding to public inquiries), and effectively generate a breadth of on-topic questions in a variety of styles. Employing generative AI, computer scientists could create algorithms and models using LLMs from emails between public users of the Blueprints website and Blueprints staff to train AI models on representative datasets in learning patterns (e.g., types of information sought by Blueprints users) and generating new data following those patterns. AI-generated parameters and ChatGPT technology could then be used to improve user search efforts by mapping information on EBPIs stored in the Blueprints database to the specific questions posed by users, with detailed audit trails tracking decisions and identifying potential biases. The primary advantage of these AI-generated parameters over Blueprints’ current structure is that navigation to find information on EBPIs within a representative dataset such as the Blueprints database would occur *incrementally* (to suit the user’s level of knowledge and experience), *flexibly* (as a bidirectional learning platform), and *interactively* (to reciprocally learn and generate responses of relevance to the user).

Second, ChatGPT software could be added onto a learning management system that connects the Blueprints 2.0 platform to other trusted scientific resources vetted by Blueprints to train AI models on how to provide community assessment and implementation support of well-tested operational protocols that ensure feasibility, fidelity, acceptability, appropriateness, and sustainability. User inquiries could then be linked from the Blueprints 2.0 platform to detailed guidance regarding how to select corresponding EBPIs that map to community needs, such as via the Communities That Care Youth Survey that identifies high levels of risk and low levels of protection in communities, with survey results and explanations provided by Communities that Care staff guiding prevention efforts around which interventions to adopt (Briney et al., 2012). These links could also connect users’ inquiries to recommendations for implementation procedures via Colorado State University Prevention Resource Center’s Implementation Toolbox (https://www.chhs.colostate.edu/prc/implementation-toolbox/) and Pennsylvania State University’s Prevention Learning Portal (https://plp.psu.edu/), which also provide human technical assistance and collectively serve as a one-stop website for prevention resources, training, and self-paced learning programs. Helping users navigate across multiple trusted prevention science platforms is a translational need unmet by Blueprints’ and other online clearinghouses’ current conception.

Third, AI software could provide guidance on research design, methods, analysis techniques, evaluation protocols, and strategies for translation to researchers partnering with community organizations and practitioners, grantees interested in satisfying evidence-based funding mandates, and consulting to policymakers (see Buckley, 2025). AI parameters could be embedded in the platform to explain criteria used to rate the evidence base of interventions and to employ tools like Evidence Gap Maps (Snilstveit et al., 2016), which are visual tools that identify what evidence exists--and where there are gaps--on the effectiveness of interventions. AI parameters could also identify interventions listed by different clearinghouses and explain how Blueprints’ ratings compare to others.

Fourth, community review boards would be established to ensure the Blueprints 2.0 platform is aligned with ethical and societal values. In addition, a protocol for stakeholder engagement would solicit feedback on the connection between Blueprints 2.0 and real-world needs. And fifth, once operational, a protocol to ensure wide-scale awareness of the resultant AI-augmented clearinghouse would be created, familiarizing potential end-users with its utility—in effect, advancing the uptake of evidence-based interventions. A rigorous, well-tested marketing methodology for this protocol will determine the resonance of messaging frameworks with different audiences for further refinement and targeting, and the construction of an effective delivery vehicle.

## Discussion

This conception for a 2.0 clearinghouse platform that is driven by human expertise and augmented by AI would fill gaps identified in the Bridgespan Report (Neuhoff et al., 2015) by: (1) using machine learning to create a “living evidence review” that improves the timeliness and comprehensiveness of evidence syntheses; (2) adding a chatbot search engine to map information on evidence-based programs to users’ specific questions and offer assessment and implementation support of operational protocols that ensure feasibility, acceptability, cultural relevance, and sustainability; (3) providing information on policies and management systems needed to exert the greatest benefits; (4) using concise language to describe evaluation criteria and provide explanations of how effectiveness ratings vary across clearinghouses; and (5) substantially increasing the reach and salience of the information provided in this “closed” AI-augmented clearinghouse platform to a broader range of constituent groups via a large-scale outreach campaign as well as targeted marketing efforts directed toward specific end-users.

The effort described in this paper can be achieved with sufficient funding and oversight, as well as by establishing community review boards and stakeholder engagement involving the expertise of practitioners, policymakers, ethicists, academics, evaluators, clearinghouse experts, implementation scientists, and computer scientists to advise on the development and deployment of the platform. And critical to ensuring its utility, input must be sought on an ongoing basis from all users working in concert with researchers and experts on the clearinghouse team. After initial outlays, money saved by implementing programs proven to reduce and prevent future problems can be used to fund research needed to develop new interventions supporting historically under-represented populations (Buckley, 2025; Buckley et al., 2025), establish effects, track outcomes, and finance more evidence-based strategies—which can contribute to more effective government operations and social spending.

### Implications

Today, we sit at an important crossroads for evidence-based decision making. The demands on online clearinghouses are greater than ever, and they must be configured to help entire fields—from public health and child welfare to education and labor/employment—track an increasingly voluminous and scientifically complex literature on the effectiveness of policy and programmatic interventions for infants, children, adolescents, young adults, seniors, families, and communities. They perform a critical function to evaluate the available evidence in terms of its rigor and relevance, given the wide range of types and quality of studies and the limited time and expertise of decision makers to assess and synthesize individual studies (Buckley, 2024).

Clearinghouses are also now being called upon by community members and practitioners to expand the evidence they review and synthesize beyond their initial focus primarily on evidence from experimental studies (Buckley et al., 2025; Newcomer et al., 2023). This development follows growing recognition of the importance of multiple forms of evidence for understanding what works, for whom, and under what conditions—and the myriad sources of innovation emerging from within communities, especially those that have long been marginalized and under-resourced. At the same time, starting in January 2025, the United States began enacting sweeping reductions to federally funded research and the evidence ecosystem writ large. These actions, which encompass significant budget cuts, mass layoffs, and structural reorganizations across key health and education agencies, are reverberating globally. Online clearinghouses therefore *must* evolve functionally not only to deliver on their purpose, but also to sustain (or for some, rebuild) their efforts. AI can conduct time-intensive clerical tasks of manually searching for and summarizing reports and repeatedly responding to emails and phone calls about where to find information on effective interventions, which would free staff up to expand and scrutinize the screening and evidence coding and offer personalized guidance on adopting, adapting, and scaling programs. As such, this proposed platform should pose minimal threats to the workforce of researchers, prevention scientists, and implementation specialists who currently power the evidence ecosystem. Rather, it would liberate personnel from the laborious work of searching databases and summarizing study results to perform the tasks for which they are trained, including investing more of their time in their own research, teaching, and service.

### Policy Benefits

The AI-augmented clearinghouse with the features outlined herein will provide an updated, comprehensive one-stop resource for information and assistance to deploy a range of evidence-based strategies. Researchers could readily access the available evidence, apply Evidence Gap Maps (Snilstveit et al., 2016) to identify the areas requiring further research, and continuously add to the database of effective interventions. Community members and organizations could identify and scale evidence-based interventions. Policymakers can readily determine the most effective policies to legislate and fund (such as programs that reduce disparities). Agencies at all levels of government and community organizations can put into practice effective and cost-saving programs. Meanwhile, platforms like Fig. 1 could be applied to other public policy areas—e.g., health care, mental health and substance use treatment, environmental concerns, economic policies, and national security—to dramatically improve the efficiency and effectiveness of a wide range of government operations.

Before allocating public and/or private funds and recommending interventions via AI-augmented systems, however, there must be adherence to a set of shared guiding principles that ensure ethical and responsible use. Transparency, accountability, fairness, ethics, inclusivity, privacy, accuracy, collaboration, accessibility, sustainability, human oversight, and adaptability—principles threaded throughout this paper—are crucial to leveraging AI for the greater good. By following these principles, the prevention science field can harness the power of AI to improve lives and livelihoods while safeguarding the values and integrity of our society.

## Supplementary Information

Below is the link to the electronic supplementary material.

ESM 1 (DOCX 28.0 KB)

## Data Availability

N/A.

## Code Availability

N/A.

## Data Availability

N/A.

## Declarations

### Open Research Statement

This study was not conducive to preregistration because it is not an empirical article; however, a pre-print (version 1–4) was posted on the Open Science Framework (https://doi.org/10.31219/osf.io/stvwa).
