# Supplementary material for: AI-Augmented Clearinghouse to Facilitate Evidence-Based Decision-Making and Social Spending: a Conceptual Framework
Source: Prev Sci. 2025 Oct 11;27(7):1203–15. doi: 10.1007/s11121-025-01848-1 (PMC13550062; doi:10.1007/s11121-025-01848-1)
Supplement: Supplementary file 1 [file 11121_2025_1848_MOESM1_ESM.docx]

**AI-Augmented Clearinghouse to Facilitate Evidence-Based Decision-Making and Social Spending: A Conceptual Framework**

**Online Supplement**

Online Resource Table 1. Blueprints 2.0: Standard of Evidence Review Criteria

| Standard | Evidence Criteria |
| --- | --- |
| Intervention Specificity* | 1. The intended participants to receive the intervention are clearly identified.    a. The relevant sociodemographic characteristics (age, gender, racial and ethnic group, socio‐economic status, urban/suburban/rural residence) are stated.    b. If the intended participants are those who have been screened based upon some characteristic(s) (e.g., a risk condition, protective factor status, a minimum level of the study outcome, or some personal or family attribute), these screening criteria and the screening process must be fully described.    c. All inclusion or exclusion criteria for program participation must be noted. 2. The intervention’s theoretical rationale/logic model is discussed explaining how the intervention is expected to have a positive effect and whether/how changes in risk or protection factors will affect outcome(s). 3. There is documentation of the intended intervention structure, content and delivery process. A clear description of the planned intervention is reported, such as what service, activity or treatment is provided, to whom, by whom, over what period, with what intensity and frequency, and in what setting. This can include the:    a. Content of the intervention.    b. Nature of the provider (e.g., social worker, teacher, psychologist, volunteer).    c. Duration of the intervention (e.g., 3 hours, 6 weeks, a school year).    d. Length of participation at each session/contact (e.g., 2 hours).    e. Frequency of sessions/contacts (e.g., daily, weekly, monthly).    f. Setting (e.g., school, community center, health clinic).    g. Mode of delivery (e.g., group‐based, one‐to‐one). In the case of a multi‐component intervention – for example, one that has components for children only, parents only, and children and parents together – it is necessary for each component to be described in these terms. |
| Evaluation Quality: Internal Validity – Risk of Bias (see Steeger et al., 2021)* | 1. Does the study have a high-quality design that is free of threats to the random assignment (e.g., consent after randomization, design confound)? 2. Does the study clearly describe the sample size at each stage of data gathering so that attrition from the randomized sample can be calculated at posttest and each follow-up? 3. Is measurement of the outcomes done independently from the delivery of the intervention, are outside raters blind to condition, and do participant reports avoid social desirability and demand bias? 4. Are the measures reliable and valid as shown by acceptable psychometric properties of the measures (e.g., interrater reliability, Cronbach’s alpha)? 5. Does the study use an intent-to-treat analysis by attempting to follow and analyze all subjects as assigned to their original condition? 6. Was the analysis done at the proper level, with multilevel statistical methods or other adjustments for clustering, when clusters rather than individuals were randomized? 7. Does the analysis control for baseline outcome measures with the use of change scores, baseline outcomes as covariates, or group-by-time interactions? 8. Does the analysis demonstrate baseline equivalence between conditions with statistical tests, effect sizes, or other measures of condition differences across all baseline sociodemographic and outcome measures? 9. Does the study demonstrate with statistical tests that any attrition beyond minimal levels is unrelated to group assignment, sociodemographic characteristics, or baseline outcomes? |
| Dissemination Readiness (see Buckley et al., 2020)* | 1. There is a clear description of the activities of the intervention. 2. The version of the intervention that met standards for evaluation quality is currently available for sites wishing to implement it. At a minimum, there is a contact person with knowledge of the intervention’s theoretical rationale and activities that were evaluated and shown to improve outcomes. Ideally, there is an up-to-date website and program materials can be ordered. 3. There is a curriculum, protocols and/or explicit implementation procedures with instructions that specify the intervention content and guide the implementation of the intervention. Ideally, this includes a manual or series of manuals specifying in detail what the intervention comprises. 4. There is an established system for monitoring implementation fidelity, including fidelity measures. Ideally, this includes measures to assess pre/post changes in outcomes. 5. There are levels of formal training or qualifications for those delivering the intervention. Ideally, this includes training provided by certified instructors. It also ideally includes ongoing technical assistance and coaching. 6. The financial resources required to deliver the intervention are specified. Ideally, there is a description of costs associated with implementing the program, including: start‐up costs; intervention implementation costs; intervention implementation support costs, such as technical assistance and training; and costs associated with fidelity monitoring and evaluation. A breakdown of cost for these separate components is identified. 7. There is reported information on the human resources required to deliver the intervention. Ideally, there is a description of staff resources needed to deliver the intervention, including required staff ratios, the required level of qualifications and skills for staff, and the amount of time they will need to allocate (to cover delivery, training, supervision, preparation and travel). |
| External Validity: CONSORT-Equity 2017 reporting of health equity in RCTs (Welch et al., 2017)** | 1. The program exclusively focuses on individuals or a population who experience socially structured disadvantage. 2. The program is culturally tailored (understanding that culture plays a significant role in shaping individuals’ behaviors, attitudes, and perceptions, the program is culturally adapted, deep-structure adapted, or culturally tailored; see Buckley et al., 2025 for further detail). 3. The sample includes participants who experience social disadvantage within the setting and context of the evaluation. 4. The sample consists of mixed racial and ethnic groups and differential impacts of the intervention are assessed, including whether analyses to estimate heterogeneity among subgroups were preregistered and powered to detect differences. |
| Cultural Relevance (AECF, 2014; Andrews et al., 2019; Samuels et al., 2009; Wiggins & Sileo, 2020) | 1. The developers and implementers understand the cultural backgrounds of the target population; they are knowledgeable about the cultural norms, traditions, and values of the group they are serving. 2. Communities and individuals from the target cultural group were central to the development and evaluation of the program. 3. Illustrations are presented in a way that participants can see themselves, their surroundings, and their values. 4. Research-informed guidance is provided to assist with appropriate cultural adaptations in aligning the program with the cultural preferences and sensitivities of the target group; this may involve modifying language, images, examples, and activities to be more culturally relevant. 5. The program is delivered in the participants’ first language 6. The content, illustrations, and language directly communicate respect for the participants’ native and/or home culture. 7. Program information is easily accessible and available in multiple formats (e.g., print, digital, oral) to accommodate different communication preferences and literacy levels. |
| Open Science (see Buckley et al., 2022)** | 1. Are any confirmatory data openly available? 2. Is any code for confirmatory tests openly available? 3. Are any research materials used to collect the confirmatory data openly available? 4. Did the authors follow a reporting guideline (e.g., CONSORT) to ensure quality and transparency in the report? 5. Was the study preregistered? 6. Outcome discrepancies:    a. Is the confirmatory hypothesis testing research clearly distinguished from the exploratory generating work in the registration?    b. Is the confirmatory hypothesis testing research clearly distinguished from the exploratory generating work in the report? 7. Are confirmatory outcomes in the registration listed in the report? |

Notes - ** indicates Blueprints current standards of evidence. ** indicates proposed expanded standards.

**References:**

Andrews, K., Parekh, J., and Peckoo, S. (2019). *How to embed a racial and ethnic equity*

*perspective in research: Practical guidance for the research process.* Child Trends Working Paper. Retrieved from: <https://cms.childtrends.org/wpcontent/uploads/2019/09/RacialEthnicEquityPerspective_ChildTrends_October2019.pdf>

Annie E. Casey Foundation, AECF (2014). *Race equity and inclusion action guide.* Annie E.

Casey Foundation: Baltimore, MD. Retrieved from: <https://www.aecf.org/resources/race-equity-and-inclusion-action-guide>

Buckley, P. R., Ebersole, C. R.*, Steeger, C. M., Michaelson, L. E., Hill, K. G., & Gardner, F. The role of clearinghouses in promoting

transparent research: A methodological study of transparency practices for preventive interventions. *Prev Sci,* 23, 787-798

Buckley, P. R., Gust, C. J., Gonzalez Coffin, S., Aikawa, S., Steeger, C. M., & Pampel, F. C. (2025). Applying an equity lens to

evidence-based preventive interventions: A systematic review of subgroup findings of experimental evaluations. *Prev Sci,* 93–106.

Buckley, P. R., Fagan, A. A., Pampel, F. C., & Hill, K. G. (2020). Making evidence-based

interventions relevant for users: A comparison of requirements for dissemination readiness across program registries. *Eval Rev, 44*(1), 51-83.

Samuels, J., Schudrich, W., & Altschul, D. (2009). *Toolkit for modifying evidence-based*

*practice to increase cultural competence.* Research Foundation for Mental Health: Orangeburg, NY. Retrieved from: <https://calmhsa.org/wp-content/uploads/2013/10/ToolkitEBP.pdf>

Steeger, C. M., Buckley, P. R., Pampel, F. C., Gust, C. J., & Hill, K. G. (2021). Common

methodological problems in randomized controlled trials of preventive interventions. *Prev Sci, 22*(8), 1159-1172.

Welch, V. A., Norheim, O. F., Jull, J., Cookson, R., Sommerfelt, H., Tugwell, P., Equity, C., &

Boston Equity, S. (2017). CONSORT-Equity 2017 extension and elaboration for better reporting of health equity in randomised trials. *BMJ, 359*, j5085.

Wiggins, M.E. & Sileo, A. (2020). *What's the role of equity in evaluation policy?* The Forum for

Youth Investment. Retrieved from: <https://forumfyi.org/wp-content/uploads/2020/02/Equity-in-Evaluation-Policy-Winter-2020.pdf>
